# Phage-based Electrochemical Sensors: A Review

**DOI:** 10.3390/mi10120855

**Published:** 2019-12-06

**Authors:** Jingting Xu, Ying Chau, Yi-kuen Lee

**Affiliations:** 1 Bioengineering Program, Department of Chemical and Biological Engineering, Hong Kong University of Science and Technology, Hong Kong, China; jxuau@connect.ust.hk (J.X.); keychau@ust.hk (Y.C.); 2 Department of Mechanical and Aerospace Engineering, Hong Kong University of Science and Technology, Hong Kong, China

**Keywords:** bacteriophage, phage display technique, immobilization, electrochemical sensor

## Abstract

Phages based electrochemical sensors have received much attention due to their high specificity, sensitivity and simplicity. Phages or bacteriophages provide natural affinity to their host bacteria cells and can serve as the recognition element for electrochemical sensors. It can also act as a tool for bacteria infection and lysis followed by detection of the released cell contents, such as enzymes and ions. In addition, possible detection of the other desired targets, such as antibodies have been demonstrated with phage display techniques. In this paper, the recent development of phage-based electrochemical sensors has been reviewed in terms of the different immobilization protocols and electrochemical detection techniques.

## 1. Introduction

Recently, bacteriophages (or phages) have attracted more and more attention in the biosensing field [1,2,3,4,5]. Phages are small viruses that lack their own metabolic machinery, instead, they use their host bacteria cells for propagation. They were discovered in the early 20th century almost simultaneously by Twort in 1915 [6] and d’Hérelle in 1917 [7]. Over the past 30 years, phages are regarded as the most abundant organisms on Earth with an estimated 10^31^ phage particles on the planet [8]. The general phage particle is composed of the enclosed nucleic acid (single-stranded DNA or RNA) and a protein envelope with different shapes [1]. They can be classified based on their morphology and the nature of their nucleic acids proposed by the International Committee on the Taxonomy of Viruses (ICTV) [9]. Generally, more than 96% of the bacteriophages are tailed (e.g., T-even phages), and the others are usually filamentous (e.g., M13 phages) and pleomorphic (able to assume different forms) [9,10,11]. After recognizing specific sites on the bacterium surface, phages can bind to their host cells by the specific receptor binding proteins (RBPs), inject DNA after depolymerization and cleavage of the bacterial outer structures and take over the host machinery to propagate new virions [12,13]. They can also be classified based on different infection and lytic cycles: one is called lytic phage that will lyse the bacteria immediately to infect the new host; the other is called lysogenic phage that will integrate their genome into the host DNA, remain dormant until stimulated for replication and propagation [1,10,14].

Based on their natural binding affinity to their hosts, bacteriophages have been widely employed for bacteria detection. The whole phage-based assays have been developed for bacteria detection involving the gene expression by reported phages inside the host cell and further detection of bioluminescence [15,16,17,18]. The fluorescent-labeled phages can also be employed for staining based detection of the target analytes upon attaching to the bacteria surface [19,20,21,22]. Detection of the released progeny phages after cell lysis could be used as an amplification method for quantitative analysis of the infected bacteria cells, serving as a promising detection technique in antibiotic susceptible testing as well [23]. This type of phage-based assays has been available as commercial kits, e.g., FASTPlaque-TB^®^ and PhageTek MB^®^, for drug resistance detection of *Mycobacterium tuberculosis* [24,25,26]. The overall accuracy of this phage amplification assay is promising but several concerns like the low sensitivity and specificity require more improvement before the widespread applicability of this technology. Studies have also been conducted concerning the phage-component based assays by taking advantage of the specific RBPs, lysins proteins and tail fibers for bacteria detection [27,28]. In addition, phages can be genetically engineered for the detection of other analytes with the phage display technique that was first reported by Smith in 1985 [29]. He demonstrated that foreign DNA fragments can be inserted into filamentous phage gene III to display a fusion peptide or protein on the phage particle, providing specific affinity for antibodies directed against the incorporated foreign sequence [29,30]. Phages expressing different peptides on the surface can be selected from a phage display library containing phage clones carrying different foreign DNA inserts, providing specific binding affinity to desired targets, including different types of antibodies and other organic analytes [30].

Over the last few decades, phage-based biosensors have been considered as a promising technology for biosensing of various analytes. It is well-known that a biosensor is a type of analytical device that can convert biological interactions into different kinds of measurable and processable signals [31]. A typical biosensor is composed of several key components: (1) bioreceptors that can specifically recognize and interact with target analytes from different samples, (2) transducers that can convert the biological responses into physically quantifiable signals such as electrochemical, optical, piezoelectric, etc. and (3) detectors that can amplify, analyze, display and record the signals [32]. In comparison with the other bioreceptors like antibodies and aptamers, bacteriophages provide several advantages in bacteria detection. Firstly, they are ubiquitous in nature and therefore they can survive under several harsh conditions. They provide high selectivity to different strains of bacteria and are harmless to humans [12]. In addition, phages can only infect and replicate within viable bacteria so they can be used to detect bacteria viability. They are also much less expensive to produce than antibodies and present a far longer shelf life [3]. Moreover, the easily genetical and chemical modification of phages makes them more competitive as they can provide more stable and controllable properties.

To date, phage-based biosensors with different detection methods have been developed, including optical [19,20,21,22], electrochemical [33,34,35], surface plasmon resonance (SPR) [36,37,38], quartz crystal microbalance (QCM) [39], magnetoelastic (ME) sensors [40,41,42,43,44], etc., among which electrochemical sensors have been noted due to their inherent advantages such as robustness, easy miniaturization, excellent detection limits, low-cost and possibility for field testing [45]. In an electrochemical biosensor, the binding of the target analytes to the sensor will result in the change of the electric properties at the interface and generate a measurable electric signal that can be used for quantitative analysis of the analytes in terms of current and potential [45,46]. Amperometric systems measure changes in the current resulted from the oxidation related to the biorecognition directly or indirectly. Usually, it provides a linear concentration-dependent response, being more sensitive and rapid compared to potentiometric biosensors [47]. In particular, impedimetric detection technique has been more and more popular due to their high sensitivity, label-free, less costly and high selectivity that will not be affected by the presence of other analytes in the samples. They are also able to provide more details about the interface between electrolyte and electrode surface, making impedimetric systems a promising solution for the increasing requirements of point of care worldwide [48].

In this paper, we focus on the recent development of phage-based electrochemical sensors for the detection of different analytes. Two main topics are covered in this review: the immobilization protocol of phages on the sensor surface and the electrochemical detection methods for bacteria and other targets. 

## 2. Phage Immobilization Protocol

To fabricate a functional phage-based biosensor, bacteriophages are usually immobilized on the sensor surface as the bio-receptors to capture target analytes. The immobilized phage particles should retain the infectivity and binding affinity to their specific host bacteria cells. In addition, the uniform and repeatable surface modification are crucial for the stability and reliability of the biosensors to obtain high sensitivity. The most widely used methods for phage immobilization include physical adsorption, chemical functionalization including covalent bonding and utilization of special interaction like biotin-avidin coupling. What is more, electric deposition based on the natural electric properties of phage has attracted more and more attention for now.

### 2.1. Physical Adsorption

The most simple and straightforward way for phage immobilization on the surface is physical adsorption. Such an approach had been used by Vodyanoy et al. to immobilize filamentous phage called clone E2 on the acoustic wave transducers for selective detection of *S. typhimurium* [49]. Quantitative monitoring with fluorescent microscopy indicated that surface coverage as around 3 × 10^10^ phages/cm^2^ could be achieved with this method for 1 h at room temperature. The effectivity of physical adsorption may be explained that the phages could be bound to the gold surface due to hydrophobic bonding, weak hydrogen bonding, van der Waals forces and the possible covalent bonding between the gold surface and the amine and thiol groups present on the surface of most phages [39,49]. This immobilization method was also utilized by the same group for phage deposition on quartz crystal microbalance (QCM) sensor [39] and surface plasmon resonance (SPR) sensor [36] for different bacteria detection.

In the work of Mejri et al., interdigitated gold electrodes were immersed in T4 phage solutions and incubated under 37 °C for 90 min in a humid chamber, followed by washing with sterile Phosphate Buffered Saline (PBS) and blocking with bovine serum albumin (BSA) to avoid non-specific surface binding. The functionalized electrode was then utilized for *E. coli* K12 detection through impedance measurements [50]. A similar approach for surface modification on SPR sensors was used by Brewster and the co-workers for detection of *L. monocytogenes* [38]. Phages Lm P4: A8 were adsorbed on the gold surface, the expressed antibody of which could interact with the surface protein on the bacterial cell surface. The same method was used by Tawil to anchor T4 and BP14 bacteriophages on SPR sensors for *E. coli* and methicillin-resistant *Staphylococcus aureus* (MRSA) detection respectively [37]. The limit of detection of the developed sensor was 10^3^ cfu/mL in less than 20 min. In addition, Chin’s group had proposed a series of phage-based magnetoelastic (ME) sensors for detection of different bacteria strains via physical adsorption [40,41,42,43,44]. They had successfully detected *S. typhimurium* with the phage-based ME sensor not only in culturing broths [51] but also in fat-free milk [43] and chicken meat [44].

Even though physical adsorption is a practical and economic immobilization method, it has several limitations, including the possible desorption during the analyte detection and the low surface coverage of deposited phages. This may result from the weak and non-specific bonding between phage and sensor surface via physical adsorption, which is largely dependent on the surface characteristics, e.g., surface charge or hydrophobicity and hydrophilicity. Therefore, the stability of the developed sensor will not be promising with this method.

### 2.2. Chemical Functionalization

Compared to physical adsorption, bacteriophages attached to chemically functionalized sensor surface provide much stronger bonding, decreasing the chance of virion detachment from the surface during measurements. In the case of electrochemical biosensors, this method is, therefore, more often used for phage immobilization. In the work of Shabani [33], the screen-printed carbon electrodes (SPEs) were electrochemically oxidized to generate carboxyl groups through chronoamperometry for 10 min by applying a potential of +2.2 V. The generated carboxyl groups were activated in the presence of acid media of 1-ethyl-3-(3-dimethylami-nopropyl) carbodiimide (EDC). The successful immobilization of T4 bacteriophages onto the surface was due to the formation of amide bonds between the protein coating (e.g., primary amines, –NH_2_) of the phage and the activated carboxylic groups (–COOH) on the SPEs. This group further improved their phage-based carbon microassays by concentrating the target bacteria cells with phage coated magnetic beads via the same chemical functionalization method [35]. Bhardwaj et al. used the same chemistry to deposit lytic bacteriophages against *S. arlettae* onto the surface of screen-printed graphene electrodes with –COOH functional groups generated from electrochemical oxidation [52]. 

**Figure 1:**
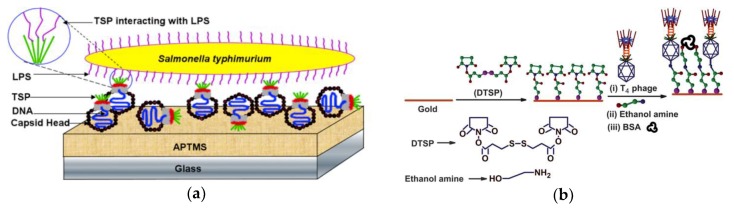
Examples of chemical functionalization methods for phage deposition: (**a**) crosslink P22 phages to the ATPMS-modified glass surface with EDC/NHS chemistry [53] and (**b**) a single-step procedure for T4 phage immobilization by using SAM of DTSP [58]. The figures were adapted with permission from [53] and [58].

Another commonly employed chemistry for surface modification was the well-established interaction between EDC and N-hydroxysulfosuccinimide (NHS). For example, in the work of Handa et al., 3-aminopropyltrimethoxysilane (APTES) was firstly used to modify the glass surface and then NHS was used along with EDC to crosslink the P22 phages to the aminosilane monolayer (Figure 1a) [53]. Hosseinidoust et al. had also proved that this EDC/NHS chemistry could be employed to covalently immobilize different types of phages (e.g., T4, MS2, PR772, etc.) onto the silanized surface of APTES-coated glass disks [54]. In addition, the EDC/NHS chemistry was adopted to generate carboxyl groups on the gold surface [55] and graphene electrode [56] for further amide coupling with the amine groups on the surface of different phages. 

Another method for chemical functionalization of phages was introduced by Niyomdecha et al., using glutaraldehyde as a crosslinker [57]. The gold electrode surface of the developed capacitive biosensor was initially electrodeposited with a polytyramine (Pty) layer, followed by the treatment of glutaraldehyde in order to activate the N-aldehyde groups of Pty layer, which would bind with the primary amino groups of the Salmonella-specific M13 bacteriophages. Arya et al. proposed a single-step procedure for immobilization of T4 bacteriophages by using self-assembly monolayer (SAM) of dithiobis (succinimidyl propionate) (DTSP; Figure 1b), the terminal succinimidyl group of which could react with primary amines present on the T4 phages [58]. Many other covalent immobilization methods based on the formation of SAMs on the sensor surface were proposed in the literature [59,60,61,62,63].

A uniform and repeatable surface modification is the key factor for the stability and reliability of the device, and it is obvious that chemical functionalization of phages onto sensor surfaces provide much more stable and denser virus layer although they are usually more time-consuming and laborious compared to physical adsorption. Singh et al. utilized sugars (dextrose and sucrose) and amino acids (histidine and cysteine) to improve the number of phages deposited at the surface via chemical bonding [60]. According to their work, surface modified with cysteine (and cysteamine) resulted in a surface coverage as 18 ± 0.15 phages/μm^2^ with glutaraldehyde as the crosslinker, representing a 37-folder increase compared to just physical adsorption on the gold surface. 

However, due to Naidoo et al.’s work, there would exist a jamming phage coverage, beyond which the specifically capturing efficiency of immobilized phages would be largely reduced [64]. Two different types of bacteriophages were studies in this work, *E. coli* T4 and *Salmonella enterica serovar Typhimurium* P22 phages. They found that the optimal planar surface density of T4 bacteriophages was 18.9 ± 0.8 phages/µm^2^ yielding the best bacteria capture density as 18.0 ± 0.3 bacteria/100 µm^2^, while the capture efficiency had decreased by at least 66.7% when beyond this value. The same trend had been observed for P22 phages with near-optimal phage coverage as 10 ± 1 phages/µm^2^. This could be explained as the increasing phage surface clustering caused some tail fibers of the deposited bacteriophages to be unavailable, therefore decreased the effective density of the special recognition proteins. Another possible reason was that both phages studied here were asymmetric, therefore, the orientation of the deposited phage would affect the accessibility of the receptors on their tail fibers, hence, control the ability to capture the target bacteria cells [54]. The random orientation would also result in the steric hindrances between deposited neighboring virions, suppressing the availability of the receptors [65].

Some researchers had used genetically modified phages to achieve this kind of orientation. Gervais et al. had reported engineering the wild type T4 bacteriophages to express a biotin-binding domain on the capsid protein with a phage display technique. The natural affinity of the biotin/streptavidin system was leveraged to immobilize these genetically biotinylated phages on a streptavidin-modified surface. This could ensure the proper orientation of the deposited phages [66]. 

### 2.3. Electric Deposition

For the phage-based electrochemical biosensors, the random orientation or the inactivation of the phages may have played an important role in the low capture efficiency of the target analytes, which will further affect the sensitivity of the sensors. It was reported that the net charge of most viruses is negative [67,68]. In the work of Baran, we know that the tail fibers of T4 bacteriophages are highly positively charged and the head structures are negatively charged, implying that the tail fibers can be electrostatically attracted to the negatively charged membrane surface of their targets for further proliferation inside the bacteria cells [69]. Anany et al. had determined the overall charge of one mutant of T4 bacteriophage composed of head and tail fibers as −5.31 ± 0.67 mV, whereas the charge was detected as 1.80 ± 0.19 mV for another mutant with only tail and tail fibers [70]. What is more, the zeta potential of T4 bacteriophages suspended in SM buffer had been detected as −9.75 ± 0.96 mV [71]. Therefore, the electric dipole properties and the negative surface charge of the bacteriophages provide the possibility of anchoring phages onto the sensor surface in a proper orientation either due to the electrostatic interaction [70,72,73] or in the presence of an external electric field [65,74]. 

**Figure 2:**
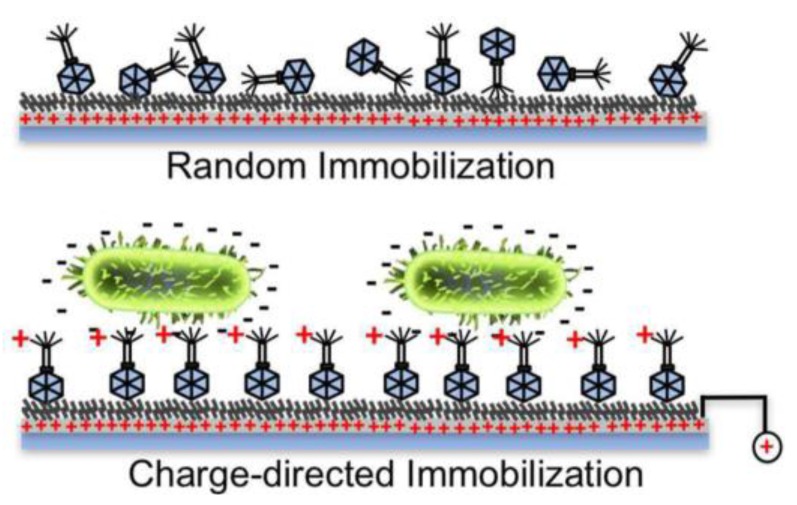
The electric deposition of T2 bacteriophages on a positively charged PEI-carbon nanotubes (CNTs). The proper orientation of phages with “head down and tail up” will increase the accessibility of receptors compared to random immobilization. The figure was adapted with permission from [73].

Anany et al. had immobilized four morphologically different phages onto the modified silica particles based on the electrostatic interaction [72]. The silica surface was modified by three different agents to modulate the surface charge from highly anionic to highly cationic. They found that the presence of ionic surfaces will enhance the number of active phages bound to the silica particles and a cationic surface will preferentially lead to the proper orientation of deposited phages with “head-down” and “tail up”. This electrostatic interaction was further utilized by this group to immobilize infective phages for *L. monocytogenes* and *E. coli* O157: H7 on the positively charged cellulose membranes, indicating a much higher phage coverage compared to unmodified membranes [70]. The similar approach was utilized by Zhou et al. to immobilize T2 bacteriophages on a glassy carbon electrode as shown in Figure 2 [73]. They functionalized the multiwall carbon nanotubes (CNTs) with polyethylenimine (PEI) to obtain a positively charged surface. A positive potential +0.5 V vs. Ag/AgCl was applied on the working electrode to deposit phages via their heads, followed by the covalent bonding with the activated PEI-modified CNTs via 1-pyrenebutanoic acid succinimidyl ester (PBSE) [73].

Another promising method to anchor the phages in the proper orientation is the application of an external electric field. Richter et al. had immobilized the T4 bacteriophages on a gold surface under the constant voltage application (10 V) for 30 min and the sensitivity of the sensor with the ordered phages provided a four-fold increase to the disordered ones [74]. In this work, they also proposed that the Debye length (*L_D_*) between the sensor’s surface and the sample solution is of great importance in the effective alignment of bacteriophages. When *L_D_* is smaller than the size of the phages, the orientation occurs almost due to electrostatic interactions while phages will be manipulated to align along the electric field lines when *L_D_* is larger than the virion size [74]. Therefore, this group later utilized an alternating electric field with the trapezoidal pulses (10 V, 16.67 kHz) in combination with chemical surface functionalization (cysteamine/glutaraldehyde and DTSP) to immobilize T4 phages [65]. Compared to the constant voltage application, the AC electric field will expand during the inter-pulse periods, increasing the exposure time of bacteriophages to the actual electric field and improving the manipulation efficiency [71]. The application of external electric field resulted in a 33-fold increase in the phage surface density compared to just chemical surface modification with DTSP and a 64-fold increase in the sensor sensitivity in comparison to physical adsorption immobilization method [65]. The combination of chemical functionalization with EDC/NHS chemistry and AC electric orientation was also performed by Xu et al. to improve the phage deposition protocol with the study of effect of Debye length as well [71]. They proposed that when the Debye length of the suspension was close to the phage size, it will provide a 26-fold increase in capture efficiency compared to physical adsorption method. These results indicate that the proper orientation of bacteriophages onto the sensor surface provides not only the higher phage coverage but also the increased accessibility of the specific receptors, which, in turn, will improve the sensor performance.

## 3. Electrochemical Detection

Electrochemical biosensors are noted for their low-cost, simplicity, high specificity and sensitivity, robustness and adaptability for field testing [45]. Electrochemical measurements are usually employed for the detection of electroactive species, based on the modulations of the electrical properties of the analytes that undergo redox reactions. Typical electrochemical methods consist of amperometric and potentiometric measurements [45,46]. The first biosensor developed by Clark and Lyons in 1962, Clark oxygen electrode, represented the simplest form of amperometric biosensors [46]. Generally, amperometric detection is based on the continuous measurement of current resulted from the oxidation or reduction of the electroactive species in a biochemical reaction when a potential is biased between the working and reference electrodes, which is usually directly proportional to the concentration of the target analytes [45,46,75]. The key advantages of this type of biosensors are their relative simplicity as well as excellent sensitivity. Limitations include the requirement of mediators and low specificity from the interference of other redox-active species. In potentiometric measurements, detection of target analytes is realized by monitoring the potential difference between working and reference electrodes upon recognition of the targets [48]. One typical potentiometric sensor is the ion-selective electrode (ISE), utilizing ion-selective membranes as the recognition elements [76]. Since potentiometric electrochemical sensors will not affect the analyte chemically, they are suitable to detection low-concentration analytes within small sample volumes [45].

In particular, impedimetric detection technique monitoring both resistance and reactance (impedance) has been more and more common due to their high sensitivity and specificity, label-free, less costly compared to other systems. Electrochemical impedance spectroscopy (EIS) has been employed to characterize different biological systems since the late 19th century [77]. With this method, the impedance spectrum is obtained over a broad frequency range when a small AC amplitude sinusoidal wave is applied. An equivalent circuit model (ECM) is commonly utilized to analyze the impedance spectra for more understanding of the electrochemical reaction and diffusion processes occurred on the electrode-electrolyte interface [78]. Conventional Randles circuit model is widely used, composing of several key components: solution resistance *R_sol_*, charge transfer resistance *R_ct_* representing the polarization happened when electrons are transferred across the interface, electric double layer capacitance *C_dl_* and Warburg impedance *W* related to the diffusion process [79]. A key advantage of impedance detection is the unrestricted measurement of interested biomolecules, with no requirements for the analyte to be an enzymatic substrate or for the formation of electroactive species as in amperometric sensing [48,80].

In this section, the phage based electrochemical sensors will be introduced, not only for the detection of different bacteria strains but also for detection of many other analytes with phage display techniques. 

### 3.1. Detection of Bacteria

Bacteriophages can be used as recognition elements for target bacteria detection due to their natural ability to capture and lyse their host bacteria cells. Therefore, the bacteriophage functionalized electrochemical sensor can be used for detection of different bacteria strains in many different samples. In addition, except to act as a bio-receptor, bacteriophages could also be used to infect and lyse the bacteria cells, followed by detection of the release intracellular contents. Impedimetric and amperometric measurements are the most commonly employed detection techniques in the research works related to phage-based electrochemical biosensors.

#### 3.1.1. Phage Based Impedimetric Sensors

Most phage-based electrochemical sensors for bacteria detection use electrochemical impedance spectroscopy (EIS) as the detection technique. With this method, the change in impedance resulted from capturing and infecting of target bacteria cells by immobilized phages on the working electrode surface can be measured. Tlili et al. proposed a label-free impedimetric biosensor for detection of *E. coli B* with EIS by covalently immobilized T4 bacteriophages on a cysteamine-modified gold surface [81]. The impedance measurements for different bacteria concentrations (10^3^–10^9^ cfu/mL) had been performed in the presence of [Fe(CN)_6_]^3−/4−^ as the redox probe. A significant increase of charge transfer resistance *R_ct_* can be observed with the increasing concentration of detected bacteria. This can be attributed to the blocking of accessible sites for redox reaction at the electrode surface resulted from the binding of bacteria cells to the immobilized T4 bacteriophages. The limit of detection of this electrochemical sensor was determined as 8 × 10^2^ cfu/mL in less than 15 min. A complementary approach was proposed in the same work after the electrochemical measurements by detecting the amplified *E. coli Tuf* gene with linear sweep voltammetry. The specific gene target was released after the target *E. coli B* cells were lysed by T4 phages and further amplified by the loop-mediated isothermal amplification (LAMP) assay. The limit of detection could be reduced to 10^2^ cfu/mL with this method in less than 1 h with a detection range of 10^2^–10^7^ cfu/mL. The same trend of *R_ct_* in terms of bacteria concentration could also be found in the other research works with the impedance measurements [52,82]. Bhardwaj et al. developed a screen-printed graphene electrode for sensitive detection of *S. arlettae* by covalently immobilizing the highly specific lytic bacteriophages against *S. arlettae* on the sensor surface [52]. EIS measurements were performed for the quantitative analysis of the target bacteria cells in the presence of the same redox couple as in Tlili’s work [81]. A linear increase in the values of *R_ct_* was observed in respect to the increasing bacteria concentration (2–2 × 10^6^ cfu/mL), based on which the limit of detection can be obtained as around 2 cfu/mL with good stability over 3 months [52]. Moghtader et al. developed a single-use pencil graphite electrode (PGE) physically coated with synthesized gold nanorods (GNRs) to increase the interfacial conductivity, which could enhance the sensitivity for impedance measurements [82]. T4 phages specific for *E. coli* K12 were then immobilized on the surface of GNRs-PGEs. The results from EIS indicated that the values of *R_ct_* would increase along with the increasing bacteria concentration for target bacteria cells with an estimated limit of detection as 10^2^ cfu/mL in 100 μL target suspension.

**Figure 3:**
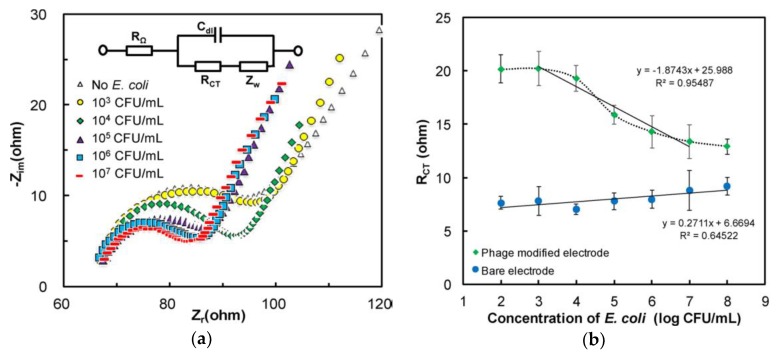
(**a**) Nyquist plot for electrochemical impedance spectroscopy (EIS) measurements of different *E. coli B* concentrations with T2 phage immobilized PEI-CNTs. The inset is the conventional Randles circuit model; (**b**) changes in the value of the charge transfer resistance *R_ct_* with the phage-modified electrode and bare electrode for different bacteria concentrations. The figure was adapted with permission from [73].

However, other researchers proposed an opposite relationship between charge transfer resistance and bacteria concentration when performing impedimetric measurements [33,50,73]. Zhou et al. developed a T2 bacteriophage-based carbon nanotube transducer for electrochemical detection of *E. coli B*. EIS measurements were used to monitor the variance in the interfacial impedance due to the specific capturing of target bacteria. When the bacteria concentration was increasing, the *R_ct_* was decreasing as shown in Figure 3, which was contrary to the previously introduced research works [52,81,82]. To understand this issue, the author also studied the interaction between phage and bacteria with a Backlight bacteria viability kit under fluorescence microscopy. An obvious decrease in the number of viable bacteria cells can be observed after around 20 min, indicating that the *E. coli B* cells had been infected and lysed by T2 phages at that time. Therefore, the decrease of *R_ct_* could be regarded as a consequence of cell lysis caused by phage infection. The cell lysis will induce more disruption of cell walls and followed by the release of intracellular components, which are usually highly mobile ionic materials (such as K^+^ and Na^+^), thus increasing the conductivity of the media near the electrode surface, in turn, decreasing the impedance detected [33,50,71]. Mejri et al. used both T4 bacteriophages and antibodies coated interdigitated microelectrodes to detect *E. coli* K12 with EIS [50]. They confirmed that compared to immunosensors, detection with phages could generate dual signals based on their intrinsic property to not only capture but also lyse target bacteria cells. Except the quantitative analysis of bacteria concentration, they also recorded the impedance variation at 233 mHz over time in the presence of *E. coli* K12 and *Lactobacillus* respectively. For *E. coli* K12, which was the host bacteria of the T4 phages, the detected impedance will firstly increase due to the attachment of bacteria cells onto the sensor surface and later decrease due to the infection and lysis of bacteria cells, which will release the internal highly conductive components, while there was no significant impedance change for *Lactobacillus*. This generation of dual signals could also help to distinguish the non-specific adsorption or the cross binding of target analytes to the sensor surface. Similar time-dependent impedance responses caused by bacteria and phage interaction had been presented by Shabani et al. [33]. They presented a functionalized screen-printed carbon electrode (SPE) microassay covalently immobilized with T4 bacteriophages for *E. coli* K12 bacteria detection [33]. Impedance measurement with EIS was employed in this work, yielding a detection limit of 10^4^ cfu/mL for 50 µL samples [33]. As discussed above, the group further improved their sensor performance by concentrating target bacteria cells from the samples with phage-coated magnetic particles [34,35]. These modified particles were then mixed with the bacteria suspension. During the measurement, the magnetic field was turned on to attract the particle-coupled bacteria cells to the sensor surface. They had successfully enhanced the sensor sensitivity by one order via the magnetic separation step with the limit of detection as 10^3^ cfu/mL for *E. coli* K12 and *Bacillus anthracis*, respectively [34,35].

Except for the whole phage-based electrochemical sensors introduced above, impedimetric biosensors utilizing specific phage components for bacteria detection have also been developed. Tolba et al. immobilized the cell wall binding domain (CBD) of bacteriophage endolysin on a gold screen-printed electrode (SPE) for specific detection of *Listeria* cells [27]. Bacteriophage endolysins are a class of bacteria cell wall peptidoglycan hydrolases that will be synthesized during the lytic cycle, mediating the lysis of host bacteria cells and further release of progeny phages [28,83]. The endolysins usually are composed of two main modular proteins, the C-terminal cell wall binding domain (CBD) and N-terminal catalytic domain [28,83]. The CBDs provide high affinity and specificity without lytic activity and could serve as the bioreceptor for target bacteria detection. With EIS measurements, Tolba et al. had utilized the CBD functionalized SPEs for detection of *Listeria* cells with a limit of detection as 1.1 × 10^4^ cfu/mL in pure culture broth and 10^5^ cfu/mL in milk, respectively [27].

#### 3.1.2. Phage Based Amperometric Sensors

In addition to the impedimetric detection, amperometric techniques are also employed in phage-based electrochemical sensors for bacteria detection. In such an approach, phages are used either as a detection probe for target bacteria cells or as a tool for specific infection and further detection of the released cell content. Li et al. proposed a promising biosensor by using the AMP magainin I as the capture probe and the phage-coated organic-inorganic hybrid nanoflowers (GOx&HRP-Cu_3_(PO_4_)_2_) as the detection probe [84]. The detection probes for *E. coli* cells were constructed by mixing the nanoflowers, gold nanoparticles (GNPs), thionine (Thi) and T4 bacteriophages, which can catalyze three cascade redox reactions in glucose working solution, serving as the signal amplification step. Differential pulse voltammetry (DPV) was employed as the electrochemical detection technique due to its enhancement of the sensitivity through minimization of the charging current [79,85], providing a very low detection limit as 1 cfu/mL and a dynamic detection range as 1.5 × 10^1^–1.5 × 10^8^ cfu/mL. The DPV measurements were also performed by Xu et al. for specific detection of *E. coli B* cells with T4 phage functionalized micro gold electrodes [71]. The phages were immobilized with the synergy of chemical functionalization and electric orientation as introduced previously. The limit of detection was obtained as 14 ± 5 cfu/mL with a wide dynamic range of 1.9 × 10^1^–1.9 × 10^8^ cfu/mL [71]. Later the group also developed phage-based extended-gate field-effect transistors (EGFETs) for target bacteria detection. The phage-deposited gold electrode was utilized as an extended gate connected to a commercial metal oxide semiconductor field-effect transistor (MOSFET). The electric characterization results indicated that the negative shift of threshold voltage was related to the net positive charge accumulation around the sensing layer, which was resulting from the release of highly mobile ionic materials (such as K^+^ and Na^+^) from cell cytoplasm when the target bacteria cell was lysed by T4 phages. The detection limit was determined as 14 ± 3 cfu/mL with a linear detection range as 10^2^–10^8^ cfu/mL [71].

As mentioned above, detection of released cell content after phage-induced lysis is another class of phage-based electrochemical sensors. Neufeld et al. proposed an electrochemical biosensor for detection of *E. coli* K12 based on the amperometric measurements of the released intracellular enzyme markers (β-D-galactosidase) [86]. A specific phage λ was used to recognize and lyse its host bacteria cells, followed by the release of the cell content, e.g., enzymes, into the suspension. The enzyme β-D-galactosidase is widely used to identify *E. coli* strains in water samples. Therefore, its activity was characterized by employing *p*-aminophenyl-β-D-galactopyranoside (β-PAPG) as the substrate with amperometric detection. The product of the reaction *p*-aminophenol (*p*-AP) was oxidized at the carbon SPEs and the measured current was recorded, providing a detection limit as low as 1 cfu/100 mL in 6–8 h. 

Wang et al. developed a similar approach for detection of *E. coli* using the T7 phages [87]. They engineered the T7 phages with *lacZ* operon encoding for β-galactosidase (β-gal), which were able to trigger the overexpression of β-gal and release a large amount of the enzyme biomarkers during the infection and lysis of *E. coli* cells. The endogenous phage-induced β-gal will then be detected with 4-aminophenyl-β-galactopyranoside (PAPG) as a substrate as described above. The product *p*-aminophenol (*p*-AP) that was proportional to the bacteria concentration could be monitored by amperometry, providing a limit of detection as 10^5^ cfu/mL in 3 h and 10^2^ cfu/mL after 7 h in aqueous samples, including drinking water, apple juice and skim milk. Neufeld et al. also proposed another method for detection of *E. coli* by combining the phage infection and amperometric detection of enzymatic activity [88]. This time, a filamentous phage M13K07 was engineered with a plasmid carrying a gene coding for the detectable bacterial enzyme, alkaline phosphatase. The reporter enzymes will be directed to the periplasmic space between the outer plasma membrane and the cell wall during the infection. Due to the porous structures of the cell wall, the substrate *p*-aminophenyl phosphate can enter easily into the periplasmic space and the product of the reaction *p*-aminophenol (*p*-AP) could diffuse out and be measured by amperometry as mentioned in their previous work [86], yielding a lower limit of detection as 1 cfu/mL in less than 3 h. This phage-based amperometric method could also be used for detection of low-concentration *Bacillus cereus* and *Mycobacterium smegmatis* [89]. The enzymes α-glucosidase and β-glucosidase were chosen as the biomarkers for *B. cereus* and *M. smegmatis*, respectively. The reporter enzymes could catalyze the *p*-amino-phenyl-α-glucopyranoside (*p*-AP-α-GLU) to *p*-aminophenol (*p*-AP) and *p*-amino-phenyl-β-glucopyranoside (*p*-AP-β-GLU) to glucose, respectively. The reaction products were then oxidized at the working electrode and the amperometric measurements were performed with a limit of detection as 10 viable cells/mL within 8 h. Except the enzymes, the released ions during the lytic cycle could also be used as markers for bacteria detection. 

Nikkhoo et al. introduced a fast and low-cost bacteria detection platform by integrating ion-selective field-effect transistors (ISFETs) with additional potassium-sensitive membrane for detection of potassium ion (K^+^) efflux resulting from infection of *E. coli* cells by T6 phages [90]. The developed platform can detect viable bacteria cells in less than 10 min with high specificity. Table 1 had summarized the aforementioned phage-based electrochemical sensors for bacteria detection.

### 3.2. Detection of Other Analytes

The applications of bacteriophage-based electrochemical sensors are not limited to bacteria detection. With the phage display techniques, different proteins or peptides providing specific binding affinity to target molecules can be displayed on the phage surface. These genetically engineered phages could be used for biosensing of many other analytes, including glucose [91,92], cancer cells [93] and antibodies [62,94,95,96,97,98,99,100,101,102].

#### 3.2.1. Glucose

Kang et al. fabricated an electrochemical sensor with bio-nanowires for sensitive glucose detection [91]. The bio-nanowire networks consisted of filamentous phages directly assembled with silver nanoparticles (Ag-NPs). The wide-type p8MMM phages were engineered to display the MMM peptide on the major coat protein pVIII that can further link with the Ag-NPs. The synthesized nanocomposites were then covalently immobilized on the active working area, followed by conjugating with the glucose oxidase (GOx). The GOx can catalyze glucose oxidation in the presence of dissolved oxygen, generating hydrogen peroxide (H_2_O_2_), which can be quantitatively measured by chronoamperometry. The proposed bio-nanowire biosensor provided sensitive detection of different glucose concentrations (10^−7^–10^−4^ M) with promising stability under different pH and temperature conditions. The filamentous phages in this work were not utilized as a bio-recognition element but as a scaffold for the attachment of the AuNPs and GOx. This approach was also utilized by Han et al. to develop an electrochemical glucose biosensor [92]. In their work, the genetically engineered M13 filamentous phages were used as the scaffolds for precise nucleation and growth of MnO_2_ crystals. The M13 phages were firstly engineered to display tetra-glutamic acid on the N-terminus of the pVIII protein, which will provide carboxyl groups for specific affinity with Mn^2+^. In the presence of NaOH, the MnO_2_ will be formed on the phage scaffolds by oxidation. The glucose biosensors were then fabricated by coating GOx onto the synthesized M13@MnO_2_ nanowires. The indirect detection of glucose can be obtained by detecting the produced H_2_O_2_ from the glucose oxidation by GOx. With chronoamperometry, the developed biosensor provided a wide linear range (5 × 10^−6^–2 × 10^−3^ M glucose) and a low detection limit as 1.8 × 10^−6^ M with good reproducibility and stability [92].

#### 3.2.2. Cancer Cells

Han et al. proposed a label-free phage-based impedimetric cytosensor for selective detection of cancer cells, taking advantage of the phage display technology [93]. The phages fused with octapeptide that were specific to the SW620 human colorectal carcinoma cells had been chosen from the "landscape phage library" and then were covalently bound to the gold sensor surface via EDC/NHS chemistry. EIS technique was utilized to characterize the performance of this biosensor for the detection of cancer cells in the presence of electroactive redox probe [Fe(CN)_6_]^3−/4−^. When the cell concentration was increasing, the values of charge transfer resistance *R_ct_* was gradually increasing as well, indicating that the attachment of the cancer cells to the sensor surface had blocked the sites for the redox probes. This cytosensor provided a wide detection range from 2 × 10^2^ to 2 × 10^8^ cell/mL and a low detection limit as 79 cells/mL with good reproducibility and high specificity.

#### 3.2.3. Antibodies

**Figure 4:**
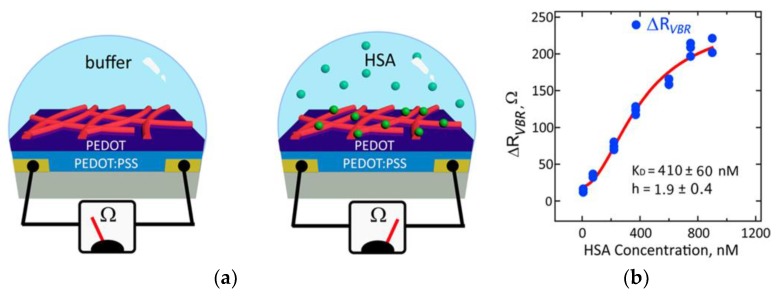
The proposed virus bioresistor (VBR) for human serum albumin (HAS) detection [99]: (**a**) compared to the buffer solution, the presence of target HSA will result in the increase of *R_VBR_* and (**b**) the sensing signal *ΔR_VBR_* was used for quantitative analysis of various HSA concentrations. The figures were adapted with permission from [99].

The phage-based electrochemical sensors used for universal bio-detection were firstly proposed by Penner’s group [62,94]. The M13 phages were genetically engineered to provide specific binding affinity for prostate-specific membrane antigen (PSMA) that was regarded as a biomarker for prostate cancer. The engineered M13 phages were then covalently immobilized onto the NHS-TE-modified electrode. The electrode with a dense virus layer was used for impedance measurements in the presence of the anti-M13 antibodies and the PSMA. Results indicated that the real part of the impedance *Z_Re_* measured from 2 to 500 kHz increased when the anti-M13 antibodies or PSMA were captured by the deposited virions. Further measurements had been carried out to study the sensitivity of the phage-based biosensors in antibody detection with EIS over a wider frequency range from 0.1 to 10^6^ Hz [94]. The results indicated that although the differences in *Z_Re_* were largest for frequencies below 1 Hz, the measured noise was also at the maximum at the low frequency. For frequency range from 4 to 140 kHz, the designed signal to noise ratio was higher, providing higher specificity. Based on the linear response of resistance change to the antibody concentration in this frequency range, a limit of detection can be obtained to be 20 nM. The group further proposed a polymer-based electrochemical sensor for detection of antibodies by incorporating the same engineered M13 bacteriophages into an array of poly(3,4-ethylene-dioxythiophene) (PEDOT) nanowires [95,96]. A bias potential 100 mV was applied across the fabricated array and the current was measured and later converted into resistance when exposed to different antibody concentrations. Binding of the specific anti-M13 antibodies to the phage-PEDOT nanowires would result in an increase in resistance along with the concentration, providing a detection limit as 20 nM [95]. The same phage-coated nanowire electrode was utilized to detect PSMA from 20 to 120 nM in high ionic buffer, yielding a detection limit as 56 nM [96]. Later, the sensitivity of the virus-PEDOT nanowires for detection of PSMA had been improved by further engineering the phage particles [97]. The density of the specific ligands for PSMA expressed on the phage surface was increased by combining the genetic coding by conventional phage display technique and the chemical synthetization by the “phage wrapping” technique. The developed biosensors provided a much lower limit of detection as 100 pM for PSMA in synthetic urine without any amplification method [97]. The group further developed virus-based electrochemical biosensors for detection of human serum albumin (HSA) [98,99]. The virus-PEDOT films were synthesized as described in their previous works [95,96] and electropolymerized onto the gold electrodes, serving as the bio-recognition element. This time, the M13 phages were engineered to selectively bind with HSA. EIS measurements were performed to record the shift in the real part of the impedance *Z_Re_* resulted from the binding of HSA to the virus-PEDOT films. The developed biosensors can detect HSA in the concentration range from 10^−7^ to 5 × 10^−6^ M within 15 min [98]. More recently, this group also proposed a virus bioresistor (VBR) that can directly transfer the binding of HSA to electric impedance signals as illustrated in Figure 4a [99]. The working principle was the same as in [98]. An equivalent circuit model was established to study the detected impedance data, including the solution resistance *R_soln_*, the resistance *R_VBR_* and capacitance *C_VBR_* of the channel. The value of *ΔR_VBR_* was used to quantitatively analyze the specific interaction of HSA in the range of 7.5–900 nM with a response time as 3–30s as shown in Figure 4b.

Phage-based electrochemical sensors were also developed by other groups for detection of different antibodies with amperometric techniques [100,101,102]. Ionescu et al. developed a T7 phage immobilized biosensor for detection of West Nile virus (WNV) IgG [100]. The wild type T7 phages were modified to display a specific peptide and used as an antigen for capturing IgG. The phages were then entrapped electrochemically in the polymeric films on the sensor surface. A secondary horseradish peroxidase (HRP)-labeled antibody was used as the detection probe. The HRP molecules will oxidase hydroquinone into quinone in the presence of H_2_O_2_. Amperometric measurements were performed by detecting the quinone reduction related to the immunoreaction between phages and antibodies, providing a wide dynamic detection range for WNV IgG dilution from 1:10 to 1:10^7^. The detection limit of this sensor was obtained as 36 pg/mL within 50 min. Interesting works had also been done by Fernández and co-workers for sensitive detection of herbicides, including molinate [101] and atrazine [102], with electrochemical immunosensors. A competitive immune-sensing system was developed for detection of molinate [101]. M13 phages displaying a peptide that mimics the target analytes were employed as tracer compounds. Molinate and the virions would compete to bind with the molinate-specific antibodies that were immobilized on magnetic beads. Anti-M13 antibodies coupled with HRP were then added for detection of phages linking to the molinate-specific antibodies. The reduction of quinine detected on the carbon screen-printed electrode with square wave voltammetry was proportional to the enzymatic activity, and in turn, inversely proportional to the molinate concentration. Compared to the conventional ELISA, this electrochemical immunosensor provided a wider linear range (4.4 × 10^−3^–10 ng/mL) and a 2500-folder lower detection limit as 0.15 ng/mL [101]. A similar approach had been employed for detection of atrazine [102]. Differently, a noncompetitive magneto-electrochemical immunosensor was developed. The M13 phages were modified to display a peptide that will specifically bind with the immunocomplex of atrazine with an anti-atrazine antibody. This sensor provided a limit of detection as 0.2 pg/mL, 200-folder lower than the competitive ELISA assays [102]. Table 2 below has compared the performance and working principles of phage-based electrochemical sensors for detection of different analytes.

## 4. Conclusions

Phage-based electrochemical sensors have received tremendous concerns for bacteria detection based on their natural affinity for specific target cells. The development of phage display technique extends the employment of wild type phages since different peptides or proteins can be displayed on the phage surface with genetically engineering, providing possibilities to bind with other target analytes, like antibodies and other small compounds. Different immobilization protocols have been proposed to functionalize bacteriophages onto the sensor surface for further detection, including physical adsorption and chemical modification. Recently, electric deposition based on the natural dipole properties of a phage has drawn more attention since it can induce the phages with proper orientation, increasing the accessibility of RBPs compared to random orientation. Attractive sensors have been developed by combining the high specific bacteriophages with low-cost and sensitive electrochemical detection techniques, the best detection limit of which has been down to even 1 cfu/mL [84]. Due to the environmental stability of phages under harsh conditions with proper storage, the developed sensors could also remain effective after a long period (over 3 months reported by Bhardwaj et al. [52]).

Although the development of phage-based electrochemical sensors over the past years seem to be impressive, there still exist many challenges in this field for real application outside the laboratory. The sensitive detection even in the presence of many other irrelevant components needs to be taken into consideration, as well as the reproducibility and stability of the phage-based sensors in the detection of environment and clinic samples. In addition, the research concerning the simultaneous detection of several bacteria strains in one sample with this method is still lacking, which is more practical for addressing bacterial infection issues in different fields including water monitoring, food industry and clinic diagnosis. What is more, the miniaturization using advanced micro/nanofabrication technology has become the new trend in the recent development of low-cost biosensors for point of care diagnostics. Therefore, integration of the developed biosensors with applicable pre-processing and post-processing techniques is of great importance for practical application on field. To date, microfluidics-based techniques have been widely integrated with biosensors for pre-concentration of bacteria cells, taking advantage of size difference [103,104], electrostatic trapping [105], gravity [106], dielectrophoresis [107,108,109] and ion-concentration polarization [110]. However, these techniques also have several limitations, for example, the limited volume of sample throughput, length of time required to complete the process and the requirement for relatively clean samples.

Despite the cost of the phages and relevant reagents are less costly compared with other bioreceptors, e.g., antibodies, their purification is still expensive and laborious from the commercial prospective, requiring more attempts to reduce the cost of the phage-based materials. Therefore, the collaboration between researchers from different fields such as biology, chemistry, engineering and electronics will help with the further improvement and development of the phage-based electrochemical sensors. 

## Figures and Tables

**Table 1:** Summary of phage-based electrochemical sensors for bacteria detection.

Phage	Target	Technique	Assay Scheme	Detection Range fu/mL	LOD cfu/mL	Ref.
T4	*E. coli K12*	Impedimetric	Carbon SPE microarray	10^2^–10^8^	10^4^	[33]
T4	*E. coli K12*	Impedimetric	Interdigitated gold microelectrode	10^4^–10^7^	10^4^	[50]
T4	*E. coli K12*	Impedimetric	Carbon SPE microarray with magnetic beads	10^2^–10^8^	10^3^	[35]
Gamma Phage	*B. anthracis*	Impedimetric	Carbon SPE microarray with magnetic beads	10^2^–10^8^	10^3^	[34]
T4	*E. coli B*	Impedimetric/LAMP	Captured bacteria with immobilized phage	10^2^–10^7^	8.0 × 10^2^	[81]
Specific phage	*S. arlettae*	Impedimetric	Graphene SPEs	2.0–2.0 × 10^6^	2	[52]
T4	*E. coli K12*	Impedimetric	Pencil graphite electrodes (PGE) with Gold nanorods (GNRs)	10^2^–10^6^	10^2^	[82]
T2	*E. coli B*	Impedimetric	PEI-functionalized carbon nanotubes	10^3^–10^7^	10^3^	[73]
CBD	*Listeria*	Impedimetric	Cell Binding Domain (CBD) functionalized SPEs	10^4^–10^9^	1.1 × 10^4^	[27]
T4	*E. coli B*	EGFET	Phage coated gold electrode as an extended gate connected to a commercial MOSFET	10^2^–10^8^	14 ± 3	[71]
T4	*E. coli*	amperometric	Organic-inorganic hybrid nanoflowers (GOx&HRP-Cu_3_(PO_4_)_2_)	1.5 × 10^1^–1.5 × 10^8^	1	[84]
Phage λ	*E. coli*	amperometric	Measurement of enzyme activity (β-galactosidase)	-	1 cfu/100 mL	[86]
M13	*E. coli TG1*	amperometric	Measurement of enzyme activity (alkaline phosphatase)	-	1	[88]
B1-7064	*B. cereus*	amperometric	Measurement of enzyme activity (α-glucosidase)	-	10	[89]
D29	*M. smegmatis*	amperometric	Measurement of enzyme activity (β-glucosidase)		10	[89]
T7	*E. coli*	amperometric	Measurement of enzyme activity (β-glucosidase)	-	10^2^	[87]

**Table 2:** Summary of phage-based electrochemical sensors for detection of other analytes.

Phage	Target	Technique	Assay Scheme	Detection Range	LOD	Ref
p8MMM	Glucose	Amperometric	Phage-AuNPs conjugated with GOx	10^−7^–10^−4^ M	-	[91]
M13	Glucose	Amperometric	M13@MnO2 nanowires coated with GOx	5 × 10^−6^–2 × 10^−3^ M	1.8 × 10^−6^ M	[92]
fd-tet	Cancer cells	Impedimetric	Engineer fd-tet phages to fuse with octapeptide	2 × 10^2^–2 × 10^8^ cells/mL	79 cells/mL	[93]
M13	PSMA	Impedimetric	NHS-TE-modified electrode	-	120 nM	[62]
M13	Anti-M13 Antibody	Impedimetric	NHS-TE-modified electrode	20–300 nM	20 nM	[94]
M13	Anti-M13 Antibody	Amperometric	M13 phages incorporated into PEDOT nanowires	20–100 nM	20 nM	[95]
M13	PSMA	Amperometric	M13 phages incorporated into PEDOT nanowires	20–120 nM	56 nM	[96]
M13	PSMA	Impedimetric	M13 phages incorporated into PEDOT nanowires	-	100 pM	[97]
M13	HSA	Impedimetric	Genetically engineered M13 phages-PEDOT films	10^−7^–5 × 10^−6^ M	100 nM	[98]
M13	HSA	Impedimetric	A virus bioresistor (VBR) with virus-PEDOT films	7.5–900 nM	7.5 nM	[99]
T7	Anti-West Nile Virus IgG	Amperometric	Engineered T7 phages entrapped in a polypyrrole film	36–3.6 × 10^7^ pg/mL	36 pg/mL	[100]
M13	Molinate	Amperometric	Competitive immune-sensing system with engineered phages as tracer	4.4 × 10^−3^–10 ng/mL	0.15 ng/mL	[101]
M13	Atrazine	Amperometric	Noncompetitive magneto-electrochemical immunosensor	10^−3^–10^4^ pg/mL	0.2 pg/mL	[102]
